# Ginsenoside Rb1 from *Panax notoginseng* Suppressed TNF-α-Induced Matrix Metalloproteinase-9 via the Suppression of Double-Strand RNA-Dependent Protein Kinase (PKR)/NF-κB Pathway

**DOI:** 10.3390/molecules27228050

**Published:** 2022-11-19

**Authors:** Wen-Tao Sun, Cindy L. H. Yang, Terry C. T. Or, Dan Luo, James C. B. Li

**Affiliations:** 1 School of Health and Life Sciences, University of Health and Rehabilitation Sciences, Qingdao 266071, China; 2 Molecular Chinese Medicine Laboratory, Li Ka Shing Faculty of Medicine, The University of Hong Kong, Hong Kong SAR, China; 3 Department of Pediatrics and Adolescent Medicine, Li Ka Shing Faculty of Medicine, The University of Hong Kong, Hong Kong SAR, China

**Keywords:** *Panax notoginseng*, matrix metalloproteinase 9, Ginsenoside Rb1, double-strand RNA-dependent protein kinase (PKR), NF-κB

## Abstract

Chronic inflammation is commonly accompanied by the stimulation of matrix metalloproteinases (MMPs) production and the degradation of the extracellular matrix. The overexpression of MMP-9 (Gelatinase B) highly participates in the progression of pathetic cardiac remodeling and liver cancer metastasis. *Panax notoginseng* (Burkill) F. H. Chen (Sanqi), a widely used traditional Chinese medicinal herb, shows myocardial protective and anti-tumor effects. In this study, we examined the inhibitory effect of different PNG extracts on tumor necrosis factor (TNF)-α-induced MMP-9 expression in cardiac myoblast H9c2 cells. Using a bioassay-guided fractionation scheme, the most active extract was fractionated by silica gel column chromatography and high-performance liquid chromatography until an active compound was obtained. The compound was identified as Ginsenoside Rb1 by nuclear magnetic resonance. Ginsenoside Rb1 inhibited TNF-α-induced MMP-9 production in both H9c2 and liver carcinoma HepG-2 cells. Interestingly, it did not affect the MMP-2 (Gelatinase A) level and the cell proliferation of the two cell lines. The inhibitory effects of Ginsenoside Rb1 may be due to its modulation of double-strand RNA-dependent protein kinase and nuclear factor kappa B signaling pathways. The results reveal the potential use of Ginsenoside Rb1 for the treatment of inflammatory and MMP-9-related cardiac remodeling and metastasis of hepatocellular carcinomas.

## 1. Introduction

Matrix metalloproteinases (MMPs) are a multigene family of calcium-dependent zinc (Zn^2+^)-containing proteinases [1]. They contribute to the initiation and development of various inflammatory diseases by inducing extracellular matrix (ECM) degradation and subsequent inflammatory processes [1,2,3,4,5]. Among different types of MMPs, MMP-9 (Gelatinase B) is one of the most complex and multifunctional members. The overexpression and dysregulation of MMP-9 are associated with various diseases [3,4,5].

Increasingly, studies indicate that MMP-9 is a potential mediator and a proximal biomarker of cardiac remodeling [6]. After post-myocardial infarction (MI), the stimulated cardiomyocytes secrete large amounts of MMP-9 as well as change the intracellular structure, myofilament and cytoskeleton [7,8,9]. In vivo, mice with targeted deletion of the MMP-9 gene showed attenuated left ventricular (LV) dilation after experimental MI [10,11]. Clinically, cardiac remodeling or ventricular remodeling often occurs in response to injuries such as ischemia, virus infection, pressure overload or volume overload, which results from the intrinsic abnormalities in the contractile machinery [12,13,14]. This process gradually impairs cardiac performance and finally leads to LV dilation and heart failure, which is associated with a high mortality rate around the world [15]. Although clinical application of pharmacological therapy and surgical approaches could release some of the symptoms of LV remodeling, more efficient and better-targeted therapies on the foundation of the remodeling are still lacking.

Moreover, MMP-9 has been suggested as a decisive contributor to promoting tumor growth and metastasis of hepatocellular carcinomas, mainly by promoting cell migration through the proteolytic degradation of the extracellular basement [16,17]. In the MMP-9 transgenic mouse model, overexpression of MMP-9 in the liver of mice significantly increased the susceptibility of transgenic animals to stimulator-induced carcinogenesis and tumor development [18]. Clinically, when tumor cells have already metastasized, conventional therapeutic strategies can’t help to improve the survival of patients with solid tumors. Nevertheless, they are frequently too toxic to the patients as they do not target tumor cell-specific molecular defects [19]. Therefore, more targeting and mechanism-based therapies are urgently needed for the control of cancer metastasis.

Physiologically, the activity of MMPs is regulated mainly at three levels: transcription, activation of the latent pro-enzymes and inhibition by their endogenous inhibitors [20]. Many broad-spectrum MMP inhibitors have been developed depending on the Zn^2+^-containing structure and the regulatory pathways of MMPs. Due to the lack of specificity, toxicity, or insufficient efficacy of these inhibitors, they exhibited serious side effects in clinical trials [21]. Although the recent development of therapeutic antibodies to MMP-9 offers a strategy to overcome some of these problems, sufficient efficacy is still lacking. Therefore, highly selective and safe MMP-9 inhibitors are urgently needed for clinical use.

*Panax notoginseng* (Burkill) F. H. Chen (PNG), or Sanqi, is a traditional Chinese medicine that has been widely used as a tonic and hemostatic drug for more than 400 years in China. More than 200 compounds have been isolated from PNG, including saponins, flavonoids, cyclopeptides, saccharides and inorganic elements [22]. Previous studies have demonstrated that PNG saponins possessed both myocardial protective and anti-tumor effects [23,24,25,26]. Although some saponins (Notoginsenoside R1, Ginsenoside Rg1, Rh1 etc.) have been reported to suppress MMP-9 activity in cancer cell lines [27,28,29], a comprehensive screening of PNG targeting MMP-9 is still lacking.

**Figure 1:**
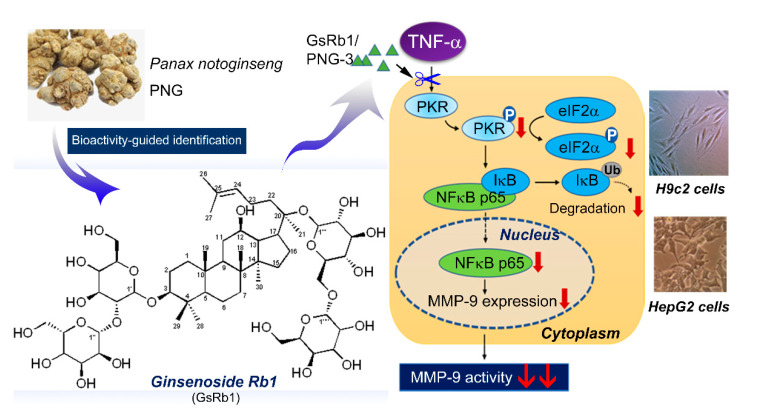
The anti-MMP-9 activity of the active fraction of PNG-3 and Ginsenoside Rb1 (GsRb1) from *Panax notoginseng* and their mechanism of action.

We extracted PNG using different extraction methods and compared their MMP-9 inhibitory effects using tumor necrosis factor (TNF)-α-treated rat cardiomyocytes (H9c2) and human hepatoma cells (HepG-2) as the bioassays. TNF-α was used as an MMP-9 inducer to mimic the chronic inflammatory condition in vitro. Using a bioassay-guided fractionation scheme, the active extract was fractionated by silica gel column chromatography and high-performance liquid chromatography until a single bioactive compound was obtained. We identified the compound as Ginsenoside Rb1 (Sanchinoside E1; Gypenoside III) using nuclear magnetic resonance. We then further delineated its mechanism of action on the cascade of double-strand RNA-dependent protein kinase (PKR)/nuclear factor kappa B (NF-κB) signaling pathways. A schematic diagram for the anti-MMP-9 activity of the active fraction of PNG-3 and Ginsenoside Rb1 (GsRb1) from PNG was illustrated in Figure 1. These findings may have clinical implications for the potential use of PNG and its bioactive molecule for alleviating MMP-9-involved inflammatory injuries, including cardiac remodeling and cancer metastasis.

## 2. Results

### 2.1. Isolation and Identification of the Bioactive Compound Using Bioactivity-Guided Approach

**Figure 2:**
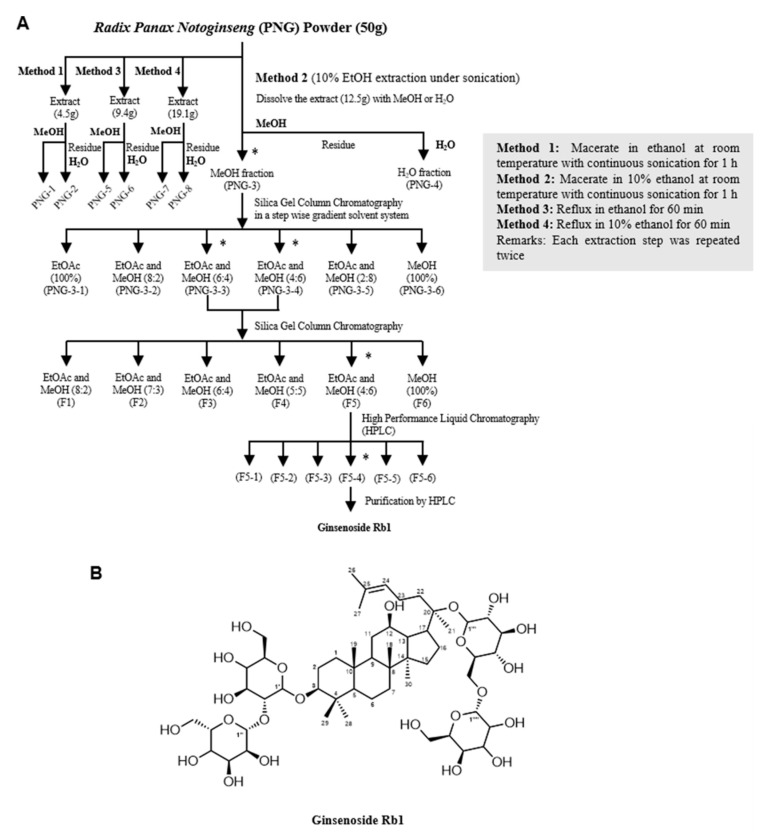
Isolation and identification of the bioactive compound Ginsenoside Rb1 (GsRb1). (**A**) The extraction scheme of GsRb1 from *Panax notoginseng* (PNG). PNG powder was first sonicated in 10% ethanol (EtOH) at room temperature. The dry extract was further successively dissolved in methanol (MeOH), and the residue was dissolved in water (H_2_O). The extracts were tested for their inhibitory effects on MMP-9 activity on TNF-α-induced H9c2 cells. The most potent extract, PNG-3, was further separated using column chromatography and reverse-phased HPLC until a single bioactive molecule was isolated. * Represents the fractions with inhibitory effect on TNF-α-induced MMP-9 activity. Other extraction methods (Method 1, 3 and 4) are also summarized. (**B**) Chemical structure of Ginsenoside Rb1.

We used different solvents (ethanol and water) and methods (sonication and reflux) to extract the components from PNG. Details of the extraction methods are summarized in Figure 2A.

The eight extracts were investigated for their inhibitory effects on the mRNA expression and activity of MMP-9 on TNF-α-induced H9c2 cells. As an endogenous mediator of inflammation and a commonly-used MMP-9 inducer [30,31], TNF-α was applied in a relatively low dosage (10 ng/mL) for a long period of time (72 h) to mimic the situation of chronic inflammation of cardiomyocytes. Results demonstrated that PNG-3 showed the most significant inhibitory effect on MMP-9 gene expression and activity on TNF-α-induced H9c2 cells (*p* < 0.0001; *p* < 0.001) (Figure S1). Besides, PNG-3 displayed no significant cytotoxicity to H9c2 cells after incubation for 72 h (Figure S2). Therefore, PNG-3 was chosen for further separation and stepwise purification.

**Figure 3:**
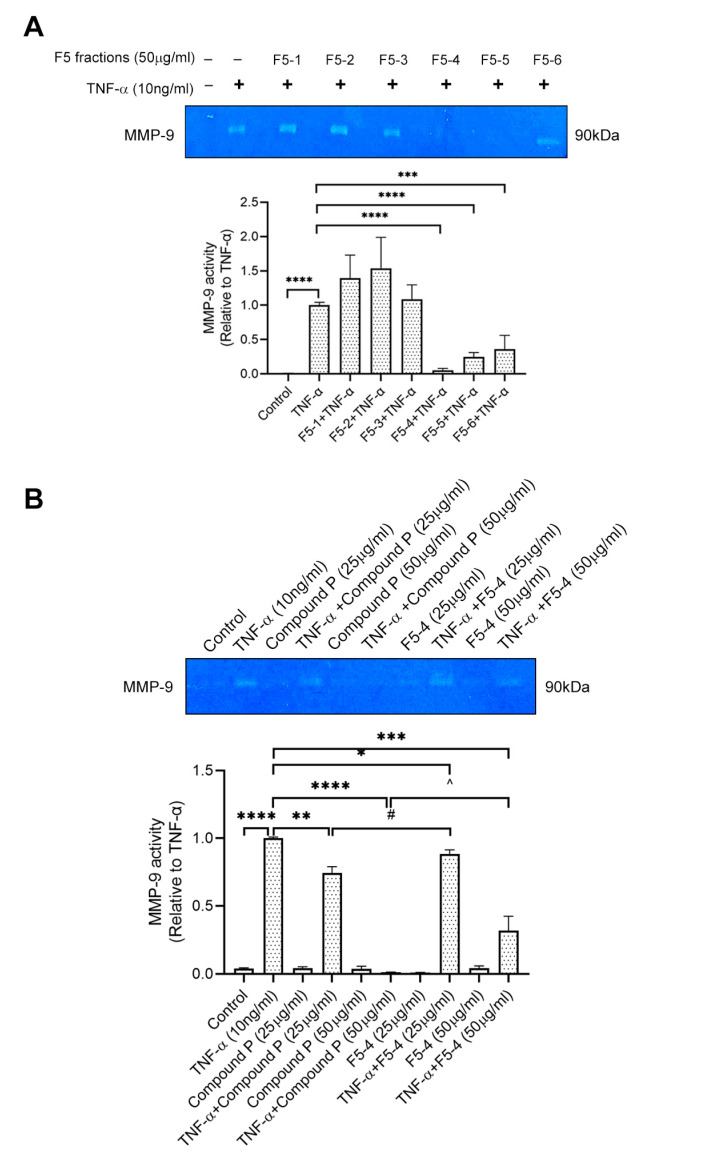
Effect of F5 subfractions (F5-1 to F5-6) and Compound P on TNF-α-induced MMP-9 activity using gelatin zymography. (**A**) H9c2 cells were seeded at 5 × 10^4^ cells/mL. After being pretreated with the six subfractions of F5 (50 μg/mL) separately for 24 h, the cells were stimulated by recombinant rat TNF-α (10 ng/mL) for another 72 h. (**B**) H9c2 cells were pretreated with compound P (25 μg/mL, 50 μg/mL) and F5-4 (25 μg/mL, 50 μg/mL) separately and then stimulated by recombinant rat TNF-α (10 ng/mL) for another 72 h. Results are shown as mean ±SD, *N* = 4. The fold induction of MMP-9 activity in H9c2 cells was normalized with that of the TNF-α-only treated cells. * *p* < 0.05, ** *p* < 0.01, *** *p* < 0.001, **** *p* < 0.0001; # *p* < 0.05 vs. TNF-α+Compound P (25 μg/mL); ^ *p* < 0.05 vs. TNF-α+Compound P (50 μg/mL).

PNG-3 was further separated into six fractions (PNG-3-1 to PNG-3-6) using column chromatography. Since both PNG-3-3 and PNG-3-4 showed significant inhibitory effects on MMP-9 activity (Figure S3A), they were combined and further separated by column chromatography. Six fractions were obtained namely F1, F2, F3, F4, F5 and F6 (Figure 2A). Among the six fractions, F5 showed significant inhibition of MMP-9 activity on TNF-α-induced H9c2 cells (Figure S3B), so it was chosen for further purification using high-performance liquid chromatography (HPLC; Figure S4). As shown in Figure 3A, three out of the six subfractions from F5 could inhibit MMP-9 activity significantly, and the fourth subfraction showed the most potent inhibitory effect, which was designed as F5-4. A single compound (compound P) with a retention time of 6.4 min and UV absorbance at 210 nm was isolated by hydrophilic interaction liquid chromatography (HILIC). Compound P and F5-4 at concentrations of 25 and 50 μg/mL showed significant MMP-9 inhibitory activity. Compared to F5-4, compound P showed a higher suppressive effect on MMP-9 activity (Figure 3B).

The ^1^H NMR spectra of compound P are shown in Figure S5A. In a comparison of the spectroscopic data with the data reported in the literature [32], the structure of compound P was elucidated as Ginsenoside Rb1 (GsRb1, C_54_H_92_O_23_, molecular weight 1109.26), and the structure is shown in Figure 2B.

### 2.2. Inhibitory Effect of Ginsenoside Rb1 on TNF-α-Induced MMP-9 Production in H9c2 and HepG-2 Cells

**Figure 4:**
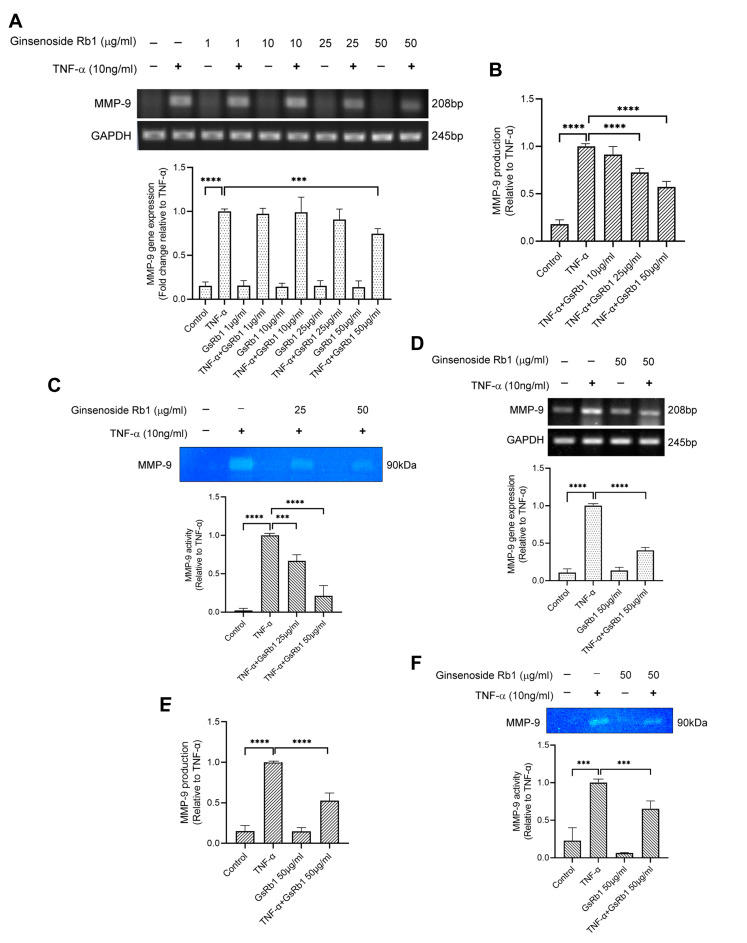
Effects of Ginsenoside Rb1 (GsRb1) on MMP-9 production in H9c2 and HepG-2 cells. (**A**) Effect of GsRb1 on TNF-α-induced MMP-9 gene expression in H9c2 cells. Cells (5 × 10^4^) were pretreated with different concentrations of GsRb1 (1, 10, 25, 50 μg/mL) for 24 h and were stimulated by recombinant rat TNF-α (10 ng/mL) for another 24 h. (**B**,**C**) Effect of GsRb1 on TNF-α-induced MMP-9 production and activity in H9c2 cells. (**D**–**F**) Effect of GsRb1 on TNF-α-induced MMP-9 gene expression, protein production, and activity in HepG-2 cells. Results are shown as mean ± SD, *N* = 4. The fold induction was normalized with that of the TNF-α-only treated cells. *** *p* < 0.001, **** *p* < 0.0001.

**Figure 5:**
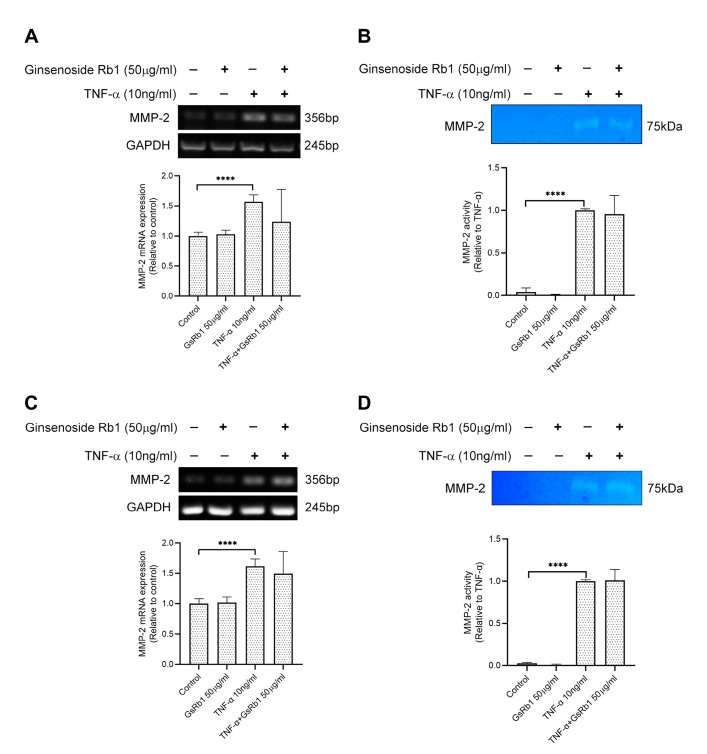
Effects of Ginsenoside Rb1 (GsRb1) on TNF-α-induced MMP-2 gene expression and activity in H9c2 and HepG-2 cells. (**A**,**B**) H9c2 cells were pretreated with GsRb1 (50 μg/mL) for 24 h. Then the cells were stimulated by recombinant rat TNF-α (10 ng/mL) for another 24 or 72 h. After the treatment, the cells were collected to examine the gene expression (**A**) and protein activity (**B**) of MMP-2 by RT-PCR and zymography. (**C**,**D**) HepG-2 cells were pretreated with GsRb1 (50 μg/mL) for 24 h. Then the cells were stimulated by recombinant rat TNF-α (10 ng/mL) for another 24 or 72 h. After the treatment, the cells were collected to examine the gene expression (**C**) and protein activity (**D**) of MMP-2. Results are shown as mean ±SD from 4 independent experiments. **** *p* < 0.0001 compared with control or TNF-α only.

After identifying Ginsenoside Rb1 from PNG, its inhibitory effects on MMP-9 expression and activity were tested in H9c2 and HepG-2 cells. In Figure 4A, TNF-α increased the mRNA expression and protein activity of MMP-9 in H9c2 cells. With the pre-treatment of GsRb1, the mRNA level, protein production and protein activity of MMP-9 were inhibited in a dose-dependent manner (Figure 4A–C). GsRb1 at a concentration of 50 μg/mL showed an obvious inhibitory effect on MMP-9 activity but without affecting the cell viability of H9c2 cells (Figure S6A). Therefore, GsRb1 at 50 μg/mL was applied in the following studies. In TNF-α-induced HepG-2 cells, GsRb1 also suppressed the MMP-9 expression and activity significantly without influencing the cell viability of HepG-2 cells (Figure 4D–F and Figure S6B). Moreover, we also examined the effect of GsRb1 on gene expression and proteinase activity of MMP-2, a homologous member of MMP-9, via PCR and gelatin zymography. Our results showed that GsRb1 has no significant inhibitory effect on the mRNA expression and activity of TNF-α-induced MMP-2 in H9c2 and HepG-2 cells (Figure 5).

### 2.3. Inhibitory Effect of Ginsenoside Rb1 on TNF-α-Induced MMP-9 Production in H9c2 and HepG-2 Cells

**Figure 6:**
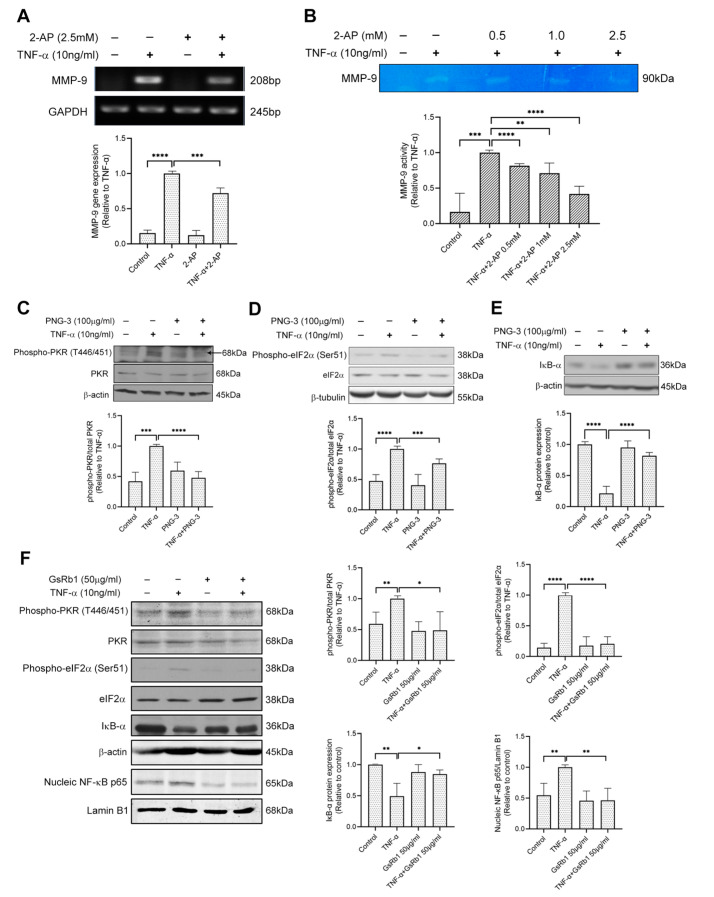
PNG-3 and Ginsenoside Rb1 (GsRb1) suppressed TNF-α-induced MMP-9 via PKR/NF-κB in H9c2 cells. (**A**) 2-AP suppressed TNF-α-induced MMP-9 gene expression in H9c2 cell. (**B**) 2-AP suppressed TNF-α-induced MMP-9 activity dose-dependently in H9c2 cell. (**C**–**E**) PNG-3 suppressed the phosphorylation of PKR and eIF-2α and preserved the expression of IκB. (**F**) GsRb1 suppressed the phosphorylation of PKR, eIF-2α and the nucleic expression of NF-κB p65, as well as preserved the expression of IκB. Results are shown as mean ± SD, *N* = 4 in each group. * *p* < 0.05, ** *p* < 0.01, *** *p* < 0.001, **** *p* < 0.0001 vs. control or TNF-α only.

To investigate whether the PKR pathway is involved in TNF-α-induced MMP-9 activation in H9c2 cells, we pretreated the cells with 2-AP, the specific inhibitor of PKR. 2-AP inhibited MMP-9 on both mRNA and protein levels (Figure 6A,B), indicating the involvement of PKR pathway on TNF-α-induced MMP-9 upregulation in H9c2 cells. Next, we examined the effects of PNG-3 (the bioactive extract) and GsRb1 on PKR signaling. Pretreatment of PNG-3 decreased the phosphorylation of PKR and eIF-2α, revealing its inhibitory effect on TNF-α-induced MMP-9 activity via the PKR pathway (Figure 6C,D). Previous studies illustrated that the NF-κB pathway was involved in MMP-9 activity induced by TNF-α [33]. Results showed that PNG-3 suppressed the degradation of IκB-α induced by TNF-α (Figure 6E,F), indicating the inhibitory effect of PNG-3 on the activation of NF-κB. Its bioactive compound, GsRb1, also decreased the phosphorylation of PKR and eIF-2α and the nuclear translocation of NF-κB p65 from the cytoplasm to the nucleus. These results revealed the inhibitory effects of PNG-3 and GsRb1 on TNF-α-induced MMP-9 activity in H9c2 cells via the regulation of the PKR/NF-κB pathway. Other signaling pathways, including ERK, p38 MAPK, JNK and PI3K, were not obviously involved (Figure S7).

**Figure 7:**
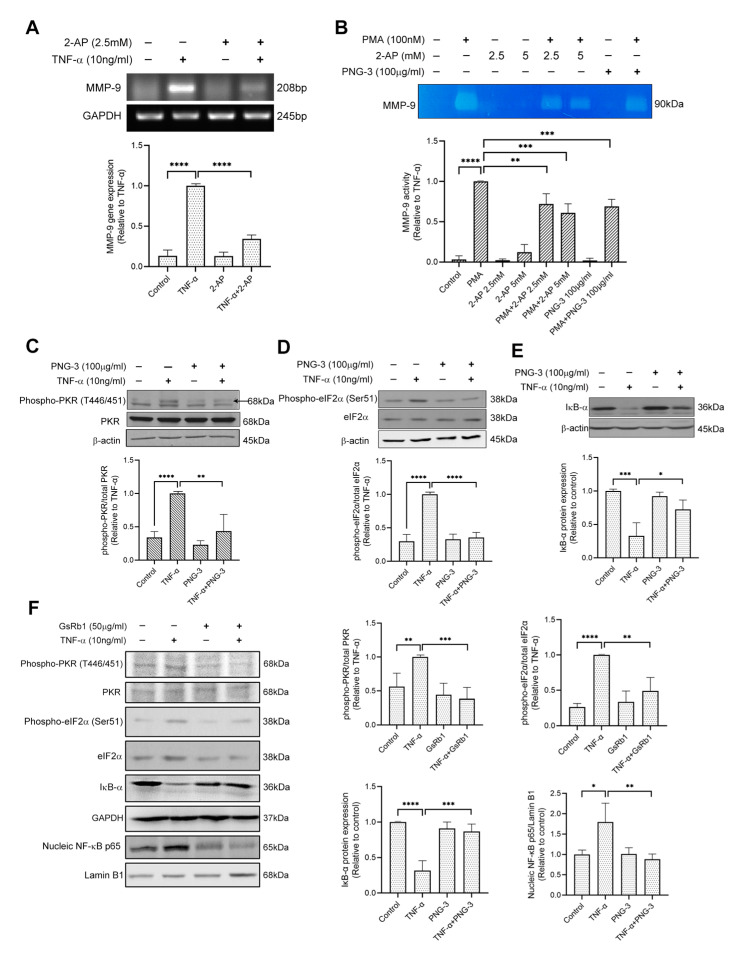
PNG-3 and Ginsenoside Rb1 (GsRb1) suppressed TNF-α/PMA-induced MMP-9 via PKR/NF-κB in HepG-2 cells. (**A**) 2-AP suppressed TNF-α-induced MMP-9 gene expression in HepG-2 cell. (**B**) 2-AP and PNG-3 suppressed PMA-induced MMP-9 activity in HepG-2 cell. (**C**–**E**) PNG-3 extract suppressed TNF-α-induced phosphorylation of PKR and eIF-2α and preserved the expression of IκB. (**F**) GsRb1 suppressed TNF-α-induced phosphorylation of PKR, eIF-2α and the nucleic expression NF-κB p65, as well as preserved the expression of IκB. Results are shown as mean ± SD, *N* = 4 in each group. * *p* < 0.05, ** *p* < 0.01, *** *p* < 0.001, **** *p* < 0.0001 vs. control or TNF-α only.

Similarly, in HepG-2 cells, 2-AP inhibited the mRNA level of MMP-9 (Figure 7A), indicating the involvement of the PKR pathway in TNF-α-induced MMP-9 upregulation in HepG-2 cells. We also treated the cell with phorbol myristate acetate (PMA), a strong stimulator of MMP-9 expression [34]. The results of gelatin zymography showed that both 2-AP and PNG-3 suppressed PMA (100 nM)-induced MMP-9 activity in HepG-2 cells (Figure 7B). Furthermore, PNG-3 and GsRb1 both showed inhibitory effects on the PKR/NF-κB pathway by inhibiting the phosphorylation of PKR and eIF-2α (Figure 7C,D,F) and the degradation of IκB-α (Figure 7E,F). GsRb1 also suppressed the nucleic expression of NF-κB p65 (Figure 7F). These results revealed the inhibitory effect of PNG-3 and GsRb1 on TNF-α-induced MMP-9 in HepG-2 cells via the regulation of the PKR/NF-κB pathway. Other signaling pathways, including ERK, p38 MAPK, JNK and PI3K, were also not obviously involved (Figure S8). This indicated that the involvement of the PKR pathway in MMP-9 production is stimulus-independent, and the general inhibitory effect of PNG-3 on MMP-9 production is via the regulation of the PKR pathway.

## 3. Discussion

*Panax notoginseng* (PNG) is one of the most commonly used herbal medicines in China and Asian countries for hemostasis and cardiovascular diseases. PNG extracts, especially the total *Panax notoginseng* saponins (PNS), have been reported to have cardioprotective and anti-tumor activities through MMP regulation [26,35,36,37]. Previous studies have demonstrated that ginsenosides Rh1, Rh2, Rb2, Rd and Rg3 showed inhibitory effects against MMP-1, MMP-2, MMP-9, TIMP-1 and TIMP-2 via the inactivation of MAPK pathways [33,38,39,40,41] (Supplementary Table S1). In the present study, we applied a bioactivity-guided identification scheme to fractionate the most effective extract (PNG-3) and isolate GsRb1 from PNG, which suppressed the expression of MMP-9 activity in H9c2 cells. GsRb1 is a major constituent in PNG. It constitutes up to 30–36% of the total PNS in PNG and is used as an internal standard for quality control of PNG [22]. It also has multiple bioactivities, as demonstrated in various disease models [23]. Although GsRb1 has been reported to attenuate the levels of many inflammatory mediators, including MMP-9, in pathetic macrophages, vascular cells and brain tissue [33,38,42,43], this is the first report to demonstrate the inhibitory effect of GsRb1 on MMP-9 activity on TNF-α-induced chronic inflammatory conditions of cardiomyocytes and hepatic cancer cells.

We then investigated how GsRb1 suppressed MMP-9 on the transcriptional level. Several intracellular signal transduction pathways are involved in MMP-9 expression, including ERK, JNK, p38 kinase, serine-threonine-protein kinase (Akt) and transforming growth factor (TGF)-β pathways [44,45,46,47]. In addition to these signaling pathways, PKR, an essential factor responding to inflammatory stresses [48], has also been reported to play a critical role in the regulation of MMP-9 [49,50]. However, the role of PKR in the MMP-9 regulation in cardiomyocyte and hepatic cancer cells and the underlying molecular mechanism is unclear. Therefore, we used 2-AP to delineate the mechanisms involved in TNF-α-induced MMP-9 activation in H9c2 and HepG-2 cells. Results showed that 2-AP is a strong PKR inhibitor that can significantly inhibit TNF-α-induced MMP-9 production (Figures S7 and S8). This study revealed that GsRb1 might function as a PKR inhibitor to inhibit TNF-α-induced MMP-9 production and activity. We further investigate the mechanism of action of GsRb1 on transcription factors. NF-κB is a well-known nuclear factor regulating MMP-9 gene expression, which normally locates in the cytoplasm bound by an endogenous inhibitory protein IκB [30,31,51]. Upon stimulation by cytokines, IκB is phosphorylated, ubiquitinated and degraded in the proteasome, leading to the translocation of NF-κB to the nucleus and promoting the transcription of MMP-9. NF-κB is also a known downstream effector of PKR [52]. Here we found that GsRb1 suppressed the degradation of IκB-α and the nucleic expression of NF-κB p65 in H9c2 and HepG-2 cells, which correlates with the transcriptional regulation of MMP-9. This is the first report that demonstrated that GsRb1 suppressed MMP-9 expression via PKR/NF-κB pathway. It broadens our understanding of the pharmacological roles of PNG and GsRb1 on TNF-α-induced chronic inflammation in cardiac cells and hepatic cancer cells.

Compared with other broad-spectrum MMP inhibitors, GsRb1 may be a potential drug candidate. In the present study, GsRb1 does not affect the cell proliferation of cardiomyocytes and liver cancer cells, which is consistent with previous reports [53,54]. The antiproliferative activities of protopanaxadiol (PPD)-type ginsenosides were inversely proportional to the number of sugar side chains in the structure [55]. As a ginsenoside with more than three sugar molecules, GsRb1 showed no significant anti-proliferative effects like the other similar compounds (ginsenoside Rc, etc.) [56]. Moreover, we found both PNG-3 and GsRb1 showed specific inhibitory effects on MMP-9 activity in H9c2 and HepG-2 cells. We also examined the expression of MMP-2 (Gelatinase A, or 72kDa Type IV Collagenase), another collagenase that is usually elevated together with MMP-9 in pathological conditions, including cardiac remodeling and cancer metastasis [57,58]. However, the results demonstrated that both PNG-3 and GsRb1 showed no significant effect on TNF-α-induced MMP-2 upregulation in H9c2 and HepG-2 cells. It may suggest a different mechanism of GsRb1 in the regulation of MMP-2 and MMP-9 in the in vitro models. So GsRb1 may work directly on the activation of PKR, which can be further confirmed by enzymatic studies. GsRb1 is one of the main constituents of the *Panax* species (Ginseng and Sanqi), numerous studies have shown its beneficial effects in cardiac remodeling via other mechanisms such as nitric oxide enhancement and mitochondria protection [59,60], and it is used as an alternative treatment of cancer metastasis in the clinic [61]. All these results revealed that GsRb1 might be a potential MMP-9-specific inhibitor with therapeutic effects on myocardial remodeling and cancer metastasis.

In light of the beneficial effects of GsRb1, a disease-specific study can be conducted. For the research of cardiac remodeling, in addition to the H9c2 cell line, the effect of PNG-3 and GsRb1 can be tested using cardiac fibroblasts, another important MMP-9 producer in cardiac diseases. We are aware that although H9c2 and HepG-2 cells are relevant and commonly used for in vitro studies for cardiac remodeling and tumor metastasis [7,8,9,17], further studies employing in vivo animal models are essential. They will help further evaluate the effects of GsRb1 in the progression of cardiac remodeling and tumor metastasis via MMP-9 inhibition, as well as confirm the efficacy and safety of the compound.

## 4. Materials and Methods

### 4.1. Chemicals and Reagents

All the chemical solvents, including ethanol (EtOH), methanol (MeOH), butanol (BuOH), ethyl acetate (EtOAc) and acetonitrile (CH_3_CN), were purchased from Tedia (USA). Gelatin, lactate dehydrogenase (LDH) assay kit, cell proliferation (MTT) kit, 2-aminopurine (2-AP, PKR inhibitor) and phorbol myristate acetate (PMA) were obtained from Sigma-Aldrich, St. Louis, MO, USA. PD 98059 (inhibitor of extracellular signal-regulated kinase (ERK)), SB 203580 (inhibitor of p38 mitogen-activated protein kinase (p38 MAPK)), SP 600125 (inhibitor of c-Jun N-terminal kinase (JNK)), and Wortmannin (inhibitor of phosphatidylinositol 3 kinase (PI3K)) were bought from Calbiochem, Germany. The recombinant rat TNF-α and rat total MMP-9 ELISA kit were bought from R&D systems, Minneapolis, MN, USA.

### 4.2. Extraction of Panax notoginseng (PNG)

The raw herbs of PNG were authenticated and obtained from PuraPharm (Nanning) Pharmaceutical Co. Ltd. They were first cut into smaller pieces and ground into powder form. PNG powder was extracted twice with 15-fold EtOH/10% EtOH (Et) under sonication (So) at room temperature for 1 h. After being concentrated by evaporation, the dried extracts were dissolved in MeOH (Me), and the undissolved residue was re-dissolved in water (H_2_O), yielding fractions of PNG-So(Et)-Me (PNG-1), PNG-So(Et)-H_2_O (PNG-2), PNG-So(10%Et)-Me (PNG-3), PNG-So(10%Et)-H_2_O (PNG-4), respectively. PNG powder was extracted twice with 15-fold EtOH/10% EtOH under reflux (Rf) for 1 h. After evaporation, the dried extracts were dissolved in MeOH, and the undissolved residue was re-dissolved in water, yielding fractions of PNG-Rf(Et)-Me (PNG-5), PNG-Rf(Et)-H_2_O (PNG-6), PNG-Rf(10%Et)-Me (PNG-7) and PNG-Rf(10%Et)-H_2_O (PNG-8), respectively. A total of 8 extracts were obtained, as shown in Figure 2A.

### 4.3. Silica Gel Column Chromatography

Using our bioactivity-guided fractionation scheme, the extract which showed an inhibitory effect on MMP-9 activity was then separated by column chromatography on an open column packed with silica gel (pore size: 35–75 μm diameter) and the extract was eluted with EtOAc with gradually increasing MeOH content from 0 to 100% (0%, 20%, 40%, 60%, 80% and 100%, each in 300 mL). Finally, 6 fractions (PNG-3-1, PNG-3-2, PNG-3-3, PNG-3-4, PNG-3-5 and PNG-3-6) were obtained. The effective fractions PNG-3-3 and PNG-3-4 were combined as PNG-3-(3+4) and then further separated using column chromatography with EtOAc with gradually increasing MeOH content from 20% to 100% (20%, 30%, 40%, 50%, 60% and 100%, each in 150 mL). Finally, 6 fractions including PNG-3-(3+4)-1, PNG-3-(3+4)-2, PNG-3-(3+4)-3, PNG-3-(3+4)-4, PNG-3-(3+4)-5 and PNG-3-(3+4)-6 were obtained.

### 4.4. High-Performance Liquid Chromatography (HPLC)

The active fraction was further separated using a Waters preparative liquid chromatography system equipped with a 1525 binary HPLC pump, 2998 photodiode array detector and Waters fraction collector III. The separation was undertaken by a reversed-phase C_18_ Lichrospher column (5µ, 250 × 4.6 mm ID), and the detection wavelength was set at 210 nm. The gradient program consisted of 2 solvents (A) water and (B) CH_3_CN at a flow of 1 mL/min as follows: 0–2 min, 5% B; 2–15 min, 5–90% B; 15–17 min, 90% B; 17–20 min, 5% B.

### 4.5. Hydrophilic Interaction Liquid Chromatography (HILIC)

The active compound was isolated and purified using an Agilent 1200 Series liquid chromatography system (Agilent, Santa Clara, CA, USA). A Water Atlantis HILIC silica (5μ, 150 × 4.6 mm ID) was used as the column, and the wavelength was set at 210 nm. The gradient program consisted of 2 solvents (A) water and (B) CH_3_CN at a flow of 1 mL/min as follows: 0–8 min, 10–50% A; 8 min, 50% A; 10 min, 10% A.

### 4.6. Structure Elucidation

The structure of the resulting pure compound was elucidated by using a Bruker 600 MHz DRX NMR spectrometer (Bruker, Billerica, MA, USA), operating at 600 MHz for ^1^H and at 150 MHz for ^13^C nuclear magnetic resonance (NMR), using methanol-*d* as the solvent. The chemical shift was referenced with respect to an internal standard (CH_3_)_4_Si (0 ppm).

### 4.7. Cell Culture

H9c2 and HepG-2 cells were bought from ATCC Cell Lines (Manassas, VA, USA). The incubation medium was Dulbecco’s modified Eagle’s minimum essential medium (DMEM), adding 10% FBS and 1% Penicillin and Streptomycin (Invitrogen Life Technologies, Carlsbad, CA, USA). The cells were cultured at 37 °C in a humidified atmosphere of 5% CO_2_ in warm air.

### 4.8. Reverse Transcription Polymerase Chain Reaction (RT-PCR)

The RT-PCR technique was used to measure the gene expression of rat MMP-9 and MMP-2. The cDNA was synthesized from total RNA with oligo(dT) primers and Superscript II reverse transcriptase (Invitrogen Life Technologies, USA). Polymerase chain reactions (PCR) were allowed to proceed for various cycles (94 °C for 30 s, melting temperature (TM) for 30 s, and 72 °C for 1 min). The following primers were used for PCR amplification: (1) Rat MMP-9 sense, 5′-AGC TGG CAG AGG ATT ACC TG-3′, antisense, 5′-TTC GAA GGT TTG GAA TTT GC-3′; (2) Rat MMP-2 sense, 5′-GGT GGC AAT GGA GAT GGA CA-3′, antisense, 5′-CCG GTC ATA ATC CTC GGT GG-3′; (3) Rat GAPDH sense 5’- ACC ACA GTC CAT GCC ATC AC-3’, antisense, 5’- TCC ACC ACC CTG TTG CTG TA-3’; (4) Human MMP-9 sense, 5′-GTA CTC GAC CTG TAC CAG CG-3′, antisense, 5′-AGA AGC CCC ACT TCT TGT CG-3′; (5) Human MMP-2 sense, 5′-CCC TGT GTC TTC CCC TTC AC-3′, antisense, 5′-ATC GTA GTT GGC TGT GGT CG-3′; (6) Human GAPDH sense 5′- AGA AGG TGG TGA AGC AGG CGT CG -3′, antisense, 5′- CCT TGG AGG CCA TGT GGG CC -3′.

### 4.9. Quantitative Real-Time Polymerase Chain Reaction (QPCR)

Quantitative real-time polymerase chain reaction (QPCR) was done to quantify the gene expression of MMP-9 for the screening of effective PNG extracts. The levels of MMP-9 mRNA were determined by QPCR (Roche 480II, Roche, Basel, Switzerland). Ribosomal RNA (18S) was used as an internal control. All the QPCR probes were obtained from the Universal Probe Library (Roche, Basel, Switzerland). All the samples were done in duplicate. The number of C_T_s of the targeted gene was normalized to that of the 18S in each sample (ΔC_T_). The mRNA expression levels of the samples were relative to the mock-treated samples (ΔΔC_T_). The relative mRNA expression of the targeted gene was calculated by 2^−ΔΔCT^ and expressed as fold induction.

### 4.10. Gelatin Zymography Assay

The proteinase activities of MMP-9 and MMP-2 were measured by gelatin zymography. 10% polyacrylamide gels copolymerized with gelatin (0.1%, G8150, Sigma) were prepared. H9c2 (5 × 10^4^ cells/mL) and HepG-2 (5 × 10^5^ cells/mL) cells were seeded at volumes of 0.5 mL in 24 well plates. After being pretreated with PNG extracts/fractions for 24 h, the cells were stimulated by recombinant rat TNF-α (10 ng/mL) for another 72 h. The supernatants were collected and loaded into each well of the same volume. Following 2 h of electrophoresis, the gels were washed with 2.5% Triton X-100 for 1 h at room temperature to remove sodium dodecyl sulfate (SDS). Gels were then incubated overnight at 37 °C in renaturing buffer (50 mM Tris-HCl, 200 mM NaCl, 5 mM CaCl_2_, pH 7.5). After incubation, the gels were stained with 0.05% Coomassie Brilliant Blue (G-250, Sigma) in a mixture of methanol: acetic acid: water (5:1:4, *v*/*v*) and destained in the same mixture without Coomassie Brilliant Blue. Gelatinolytic activities were detected as transparent bands against the dark blue background. Zymograms were digitally scanned, and band intensities were quantified using GelQuantNET software V 1.7.8.

### 4.11. Western Blot Analysis

The whole-cell protein of H9c2 and HepG-2 was extracted, fractionated, and electro-transferred to polyvinylidene difluoride (PVDF) membrane (Thermo Fisher Scientific, Waltham, MA, USA) for detection of the expression of target proteins. In brief, cells were lysed in a lysis buffer containing protease and phosphatase inhibitor cocktail (Roche Diagnostic, Basel, Switzerland) and centrifuged to collect protein. The protein extract was then fractionated by a denaturing 8% sodium-dodecyl-sulfate polyacrylamide gel electrophoresis (30 μg per lane) for 90 min at 120 V and electro-transferred to PVDF membrane for 90 min at 100 V. The membrane was blocked with 5% non-fat milk/TBST for 1 h at room temperature and incubated in 5% non-fat milk/TBST overnight at 4 °C with specific primary antibody targeting PKR (1:1000 dilution, Cell signaling technology, CST, Danvers, MA, USA), phosphor-PKR (1:1000, CST), eukaryotic initiation factor 2α (eIF-2α, 1:1000, CST), phosphor-eIF-2α (1:1000, CST), JNK (1:1000, CST), phosphor-JNK (1:1000, CST), NF-κB (1:500, CST) and inhibitory kappa B α (IκB-α, 1:500, CST) by western blotting, with Lamin B1 (1:2000, Abcam, Cambridge, UK) β-actin (1:2500, CST) and β-tubulin (1:2500, Abcam) used as nuclear and cytoplasmic loading control respectively. Anti-rabbit and anti-mouse IgG HRP-conjugated secondary antibodies were purchased from BD Sciences (San Diego, CA, USA), while anti-goat IgG HRP-conjugated secondary antibodies were purchased from ZYMED Laboratory Inc. (South San Francisco, CA, USA).

### 4.12. Protein Expression at Subcellular Level

Nuclear and cytoplasmic fractions of H9c2 and HepG-2 cells were separated by NE-PER Nuclear and Cytoplasmic Extraction Reagents (Thermo Fisher Scientific, Waltham, MA, USA). The details of the method were described in our previous study [62]. Cells were harvested with trypsin-EDTA (Thermo Fisher Scientific, Waltham, MA, USA) and centrifuged at 500 × g for 5 min to collect the cell pellet. After washing, ice-cold cytoplasmic extraction reagents I and II were added to the cell pellet in a proportion of 200:11 (μL). The supernatant was collected as cytoplasmic lysate after 5-min centrifugation at 16,000× *g*. A nucleic extraction reagent (100 μL) was then added to the precipitate and centrifuged at 16,000× *g* for another 10 min to collect the supernatant, the nuclear lysate. Proteins from nuclear and cytoplasmic preparations were examined for NF-κB expression by western blotting, with Lamin B1 (1:2000, Abcam) used as a nuclear loading control, respectively.

### 4.13. MTT Assay for Cell Viability

The influence of PNG extracts and a single compound on cell viability was assessed using an MTT assay. After the treatment, 0.5 mg/mL MTT solution (Sigma Aldrich) was used to incubate the cells for 1h at 37 °C. Then, 200 μL isopropyl alcohol (IPP) was added after the medium was discarded. After 10 min of incubation, the absorbance was measured at 570 nm by using a microplate reader (BioRad, Hercules, CA, USA).

### 4.14. LDH Assay for Cytotoxicity

A lactate dehydrogenase (LDH) assay was used to test the cytotoxicity of PNG extracts on H9c2 cells. H9c2 cells were seeded at 5 × 10^4^ cells/mL in the volume of 0.5 mL in 24 well plates. After the treatment, the cell supernatants were collected and added to the 96-well plate in 50 μL/well. Then the working reaction mixture was added to 100 μL/well. After 30 min of incubation and the addition of stop solution, the absorbance was measured at 490 nm by using a microplate reader (BioRad).

### 4.15. Enzyme-Linked Immunosorbent Assay (ELISA)

To investigate the inhibitory effect of extract PNG-3 and fraction Compound P on MMP-9 productions in TNF-α induced H9c2 cells, the total rat MMP-9 ELISA kit was used for the test. H9c2 cells were seeded at 5 × 10^4^ cells/mL in the volume of 0.5 mL in 24 well plates. After being pretreated with the extract/fraction for 24 h, the cells were stimulated by recombinant rat TNF-α (10 ng/mL) for another 48 h. Then the supernatants were collected, and MMP-9 levels were determined by ELISA according to the manufacturer’s instructions (R&D Systems, Minneapolis, MN, USA).

### 4.16. Statistical Analysis

To determine the statistical significance between the experimental groups, Student’s *t*-test was used. All experimental data are expressed as the mean value ± standard deviation. A *p*-value < 0.05 was used as an indicator of statistical significance.

## 5. Conclusions

In the present study, (1) We isolated and identified GsRb1 as an MMP-9 inhibitor from PNG using a bioassay-guided identification scheme; (2) We demonstrated that PNG-3 extract and GsRb1 suppressed TNF-α-induced MMP-9 mRNA level and activity in H9c2 and HepG-2 cells; (3) We revealed that PNG-3 extract and GsRb1 attenuate MMP-9 production via inhibiting the PKR/NF-κB pathway. The results indicate that GsRb1 is a potential drug candidate with high specificity/selectivity on MMP-9 inhibition and inflammatory alleviation, especially for cardiac remodeling and hepatocellular carcinoma metastasis.

## Data Availability

Data is contained within the article.

## Supplementary Materials

The following supporting information can be downloaded at: https://www.mdpi.com/article/10.3390/molecules27228050/s1, Figure S1: The effect of different PNG crude extracts on the mRNA expression and activity of MMP-9 in TNF-α-induced H9c2 cells. Figure S2: The effect of different PNG crude extracts on the cytotoxicity and cell viability of H9c2 cells. Figure S3: The effect of different fractions separated from PNG-3 and PNG-3-(3+4) on the activity of MMP-9 in TNF-α-induced H9c2 cells. Figure S4: Fractionation of F5 using high-performance liquid chromatography (HPLC). Figure S5: The ^1^H (a) and ^13^C (b) Nuclear Magnetic Resonance (NMR) Spectrum of Compound P. Figure S6: Effects of GsRb1 on the cell viability of H9c2 (a) and HepG-2 (b) cells. Figure S7: Underlying mechanism involved in TNF-α-indued MMP-9 activation in H9c2 cells. Figure S8: Underlying mechanism involved in TNF-α-indued MMP-9 activation in HepG-2 cells. Table S1: Summary of MMP-inhibitory saponins in *Panax notoginseng*. Citation of refs [29,33,63,64,65,66,67,68,69,70,71,72,73,74,75,76,77,78,79].

## Sample Availability

Samples of the compounds are not available from the authors.
