# Supplementary material for: Ginsenoside Rb1 from Panax notoginseng Suppressed TNF-α-Induced Matrix Metalloproteinase-9 via the Suppression of Double-Strand RNA-Dependent Protein Kinase (PKR)/NF-κB Pathway
Source: Molecules. 2022 Nov 19;27(22):8050. doi: 10.3390/molecules27228050 (PMC9692425; doi:10.3390/molecules27228050)
Supplement: Supplementary file 1 [file molecules-27-08050-s001.zip › molecules-2020427-Supplemental Materials.pdf]

SUPPLEMENTARY MATERIAL. for Article Entitled:

Ginsenoside Rb1 from *Panax notoginseng* Suppressed TNF-α-Induced Matrix Metalloproteinase-9 via the Suppression of Double-Strand RNA-Dependent Protein Kinase (PKR)/NF-κB Pathway

Wen-Tao Sun[1*], Cindy L H Yang[2], Terry C T Or[2], Dan Luo[2], James C B Li[3*]

1 School of Health and Life Sciences, University of Health and Rehabilitation Sciences, Qingdao 266071, China

2 Molecular Chinese Medicine Laboratory, Li Ka Shing Faculty of Medicine, The University of Hong Kong, Hong Kong SAR, China

3 Department of Pediatrics and Adolescent Medicine, Li Ka Shing Faculty of Medicine, The University of Hong Kong, Hong Kong SAR, China

* Correspondence: sunwt@uor.edu.cn (W.-T.S.); jameslihku@gmail.com (J.C.B.L.)

This file includes Supporting Information

Supplemental data Original NMR data of compound P (Ginsenoside Rb1) *DOI: 10.6084/m9.figshare.20660709*

Supplemental Figures

Figure S1. The effect of different PNG crude extracts on the mRNA expression and activity of MMP-9 in TNF-α-induced H9c2 cells.

Figure S2. The effect of different PNG crude extracts on the cytotoxicity and cell viability of H9c2 cells

Figure S3. The effect of different fractions separated from PNG-3 and PNG-3-(3+4) on the activity of MMP-9 in TNF-α-induced H9c2 cells.

Figure S4. Fractionation of F5 using high performance liquid chromatography (HPLC).

Figure S5: The ^{1}H (a) and ^{13}C (b) Nuclear Magnetic Resonance (NMR) Spectrum of Compound P.

Figure S6: Effects of GsRb1 on the cell viability of H9c2 (a) and HepG-2 (b) cells.

Figure S7: Underlying mechanism involved in TNF-α-indued MMP-9 activation in H9c2 cells.

Figure S8: Underlying mechanism involved in TNF-α-indued MMP-9 activation in HepG-2 cells.

Supplemental Table S1

Supplemental References

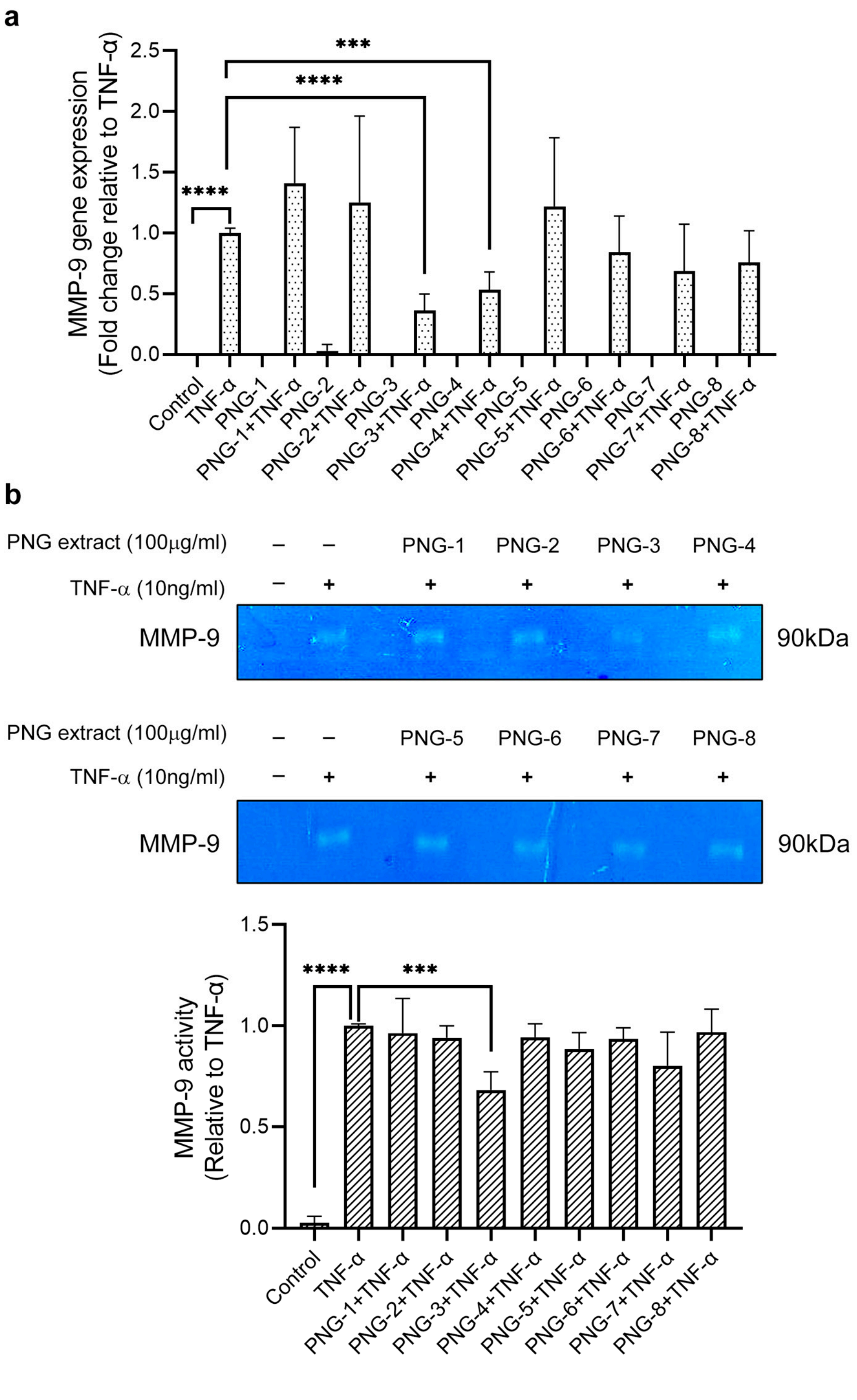

Figure S1: The effect of PNG extracts on the mRNA expression and activity of MMP-9 in TNF-α-induced H9c2 cells. H9c2 cells (5 x 10^4) were treated with 10 ng/ml TNF-α for 72 hr, in the presence of either 0.1% DMSO or 100 µg/ml PNG extracts. **a.** The mRNA expression of MMP-9 was measured by real-time PCR. **b.** The activity of MMP-9 was measured using zymography. Results were shown as mean ± SD, N=4. *** p<0.001, **** p<0.0001 compared with TNF-α only.

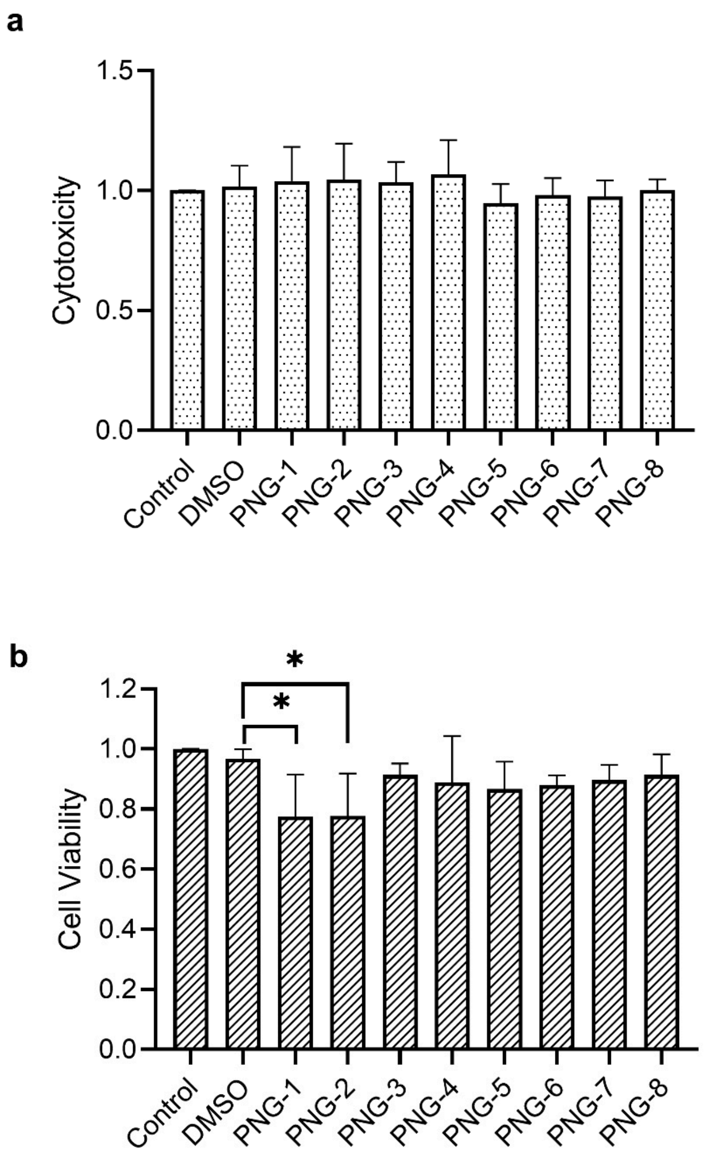

Figure S2: The effect of PNG extracts on the cytotoxicity and cell viability of H9c2 cells. a. H9c2 (5 x 10^4) were treated with 100 μg/ml PNG extracts for 72 hr. The cell death was examined by LDH assays. **b.** The cell viability was determined by MTT assays. Results are shown as mean ± SD, N=4, * p<0.05 compared with DMSO (0.1%), the solvent of PNG extracts.

a

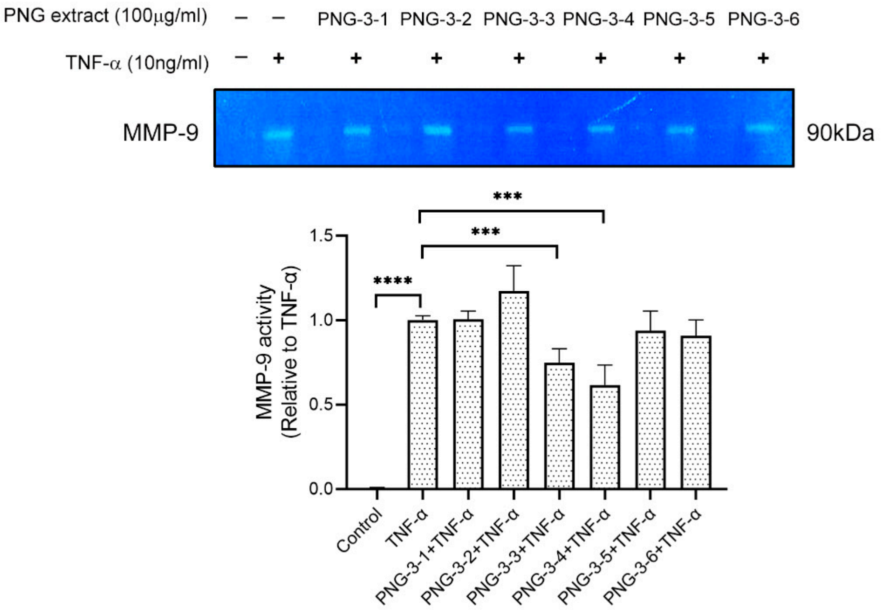

b

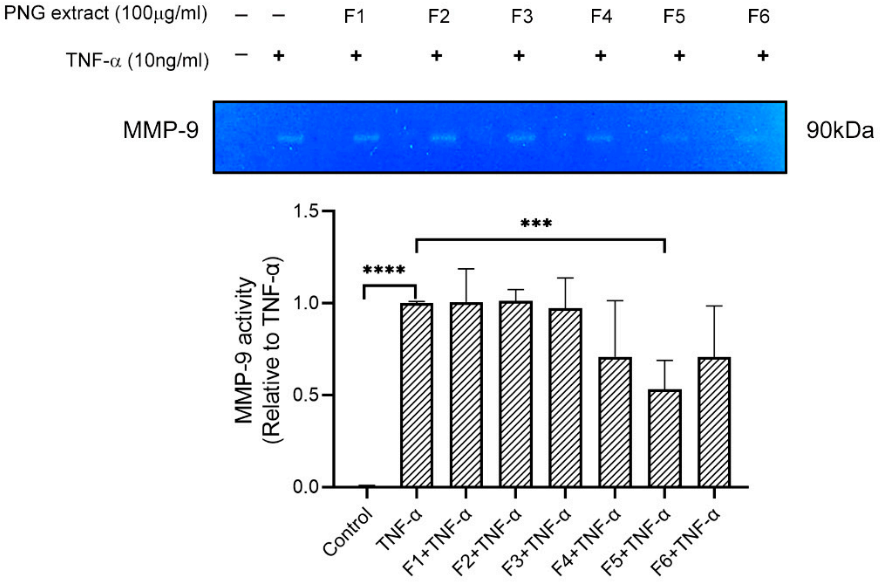

Figure S3: The effect of fractions separated from PNG-3 and PNG-3-(3+4) on the activity of MMP-9 in TNF-α-induced H9c2 cells. H9c2 cells (5×10^4) were treated with 10 ng/ml TNF-α for 72 hr, in the presence of either 0.001% DMSO or 100 μg/ml fractions. The activities of MMP-9 were measured using zymography. Results were shown as mean ± SD, N=4. *** p<0.001, **** p<0.0001 compared with TNF-α only.

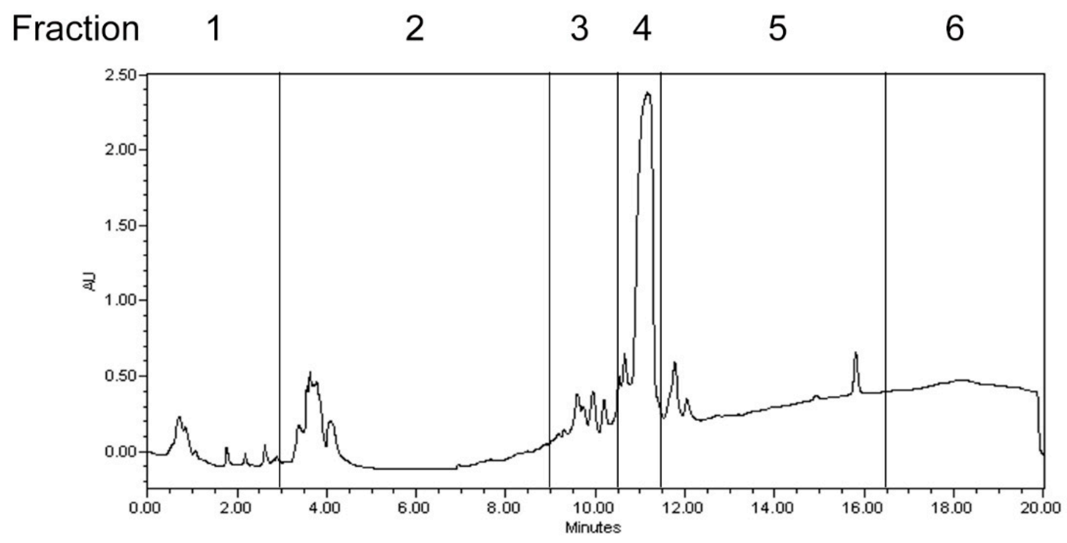

Figure S4: Fractionation of F5 using high performance liquid chromatography (HPLC). The separation was done using a reversed-phase C18 Lichrospher column (5u, 250×4.6mm ID) and the detection wavelength was set at 210nm. The gradient program consisted of two solvents (A) water and (B) CH_3CN at a flow of 1ml/min as follows: 0-2 min, 5% B; 2-15 min, 5-90% B; 15-17 min, 90% B; 17-20 min, 5% B. F5 was further separated into 6 subfractions using the same chromatographic conditions as above. The fractionation profile was depending on the retention time of F5.

a

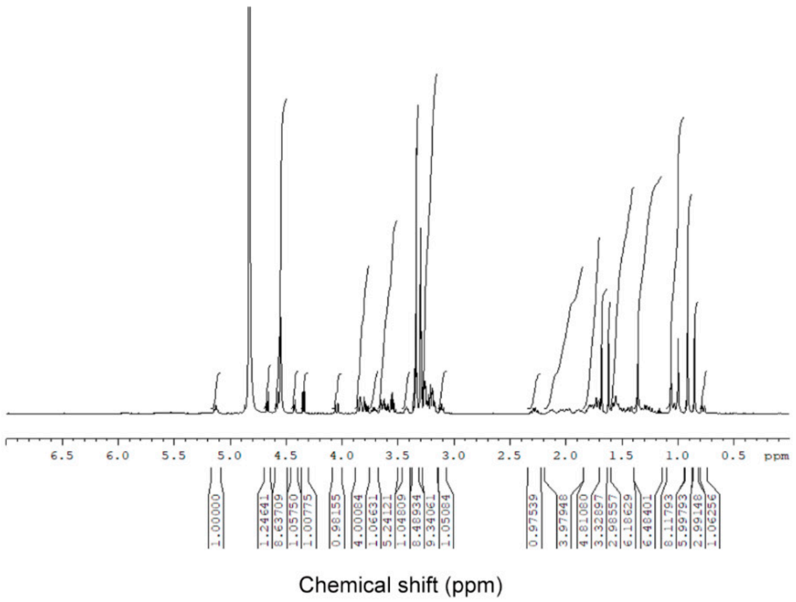

b

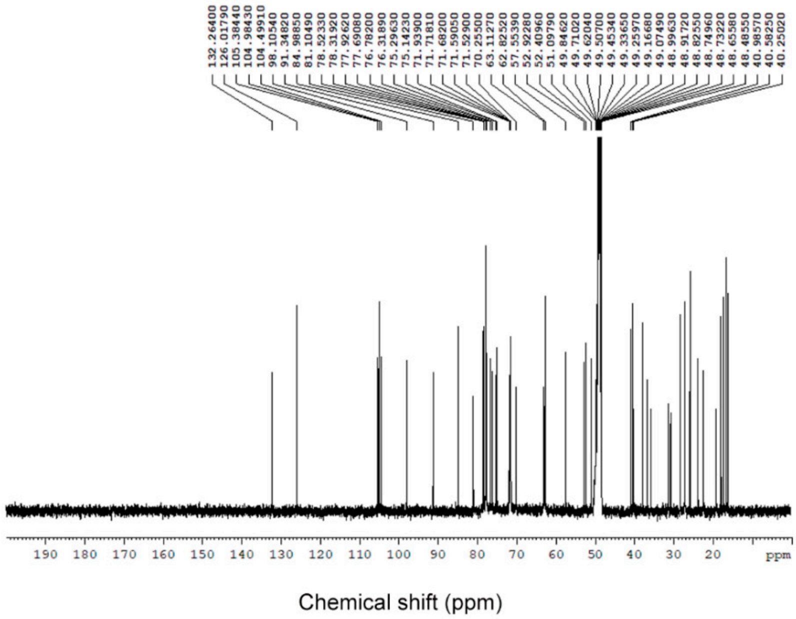

Figure S5: The ^{1}H (a) and ^{13}C (b) Nuclear Magnetic Resonance (NMR) Spectrum of Compound P. The compound was analyzed by a Bruker 600MHz PRX NMR spectrometer, operating at 600MHz for ^{1}H and 150MHz for ^{13}C NMR. Methanol-*d* was used as the solvent. The ^{13}C NMR (Methanol-*d*, 150MHz) spectra of compound P showed signals at δ 40.2 (t, C-1), 27.2 (t, C-2), 91.3 (d, C-3), 40.6 (s, C-4), 57.6 (d, C-5), 19.2 (t, C-6), 35.8 (t, C-7), 40.9 (s, C-8), 51.1 (d, C-9), 37.9 (s, C-10), 30.8 (t, C-11), 71.5 (d, C-12), 49.7 (d, C-13), 52.4 (s, C-14), 31.5 (t, C-15), 27.2 (t, C-16), 52.9 (d, C-17), 16.69 (q, C-18), 16.3 (q, C-19), 84.9 (s, C-20), 22.5 (q, C-21), 36.8 (t, C-22), 23.9 (t, C-23), 126.0 (d, C-24), 132.3 (s, C-25), 25.9 (q, C-26), 18.0 (q, C-27), 28.4 (q, C-28), 16.74 (q, C-29), 17.3 (q, C-30), 104.9 (d, C-1’), 81.0 (d, C-2’), 77.9 (d, C-3’) , 71.6 (d, C-4’), 78.3 (d, C-5’), 62.8 (t, C-6’), 105.4 (d, C-1”), 77.7 (d, 2”), 78.5 (d, 3”), 71.7 (d, 4”), 77.9 (d, 5”), 63.1 (t, 6”), 98.1 (d, 1’”), 75.1 (d, 2’”), 78.5 (d, 3’”), 71.7 (d, 4’”), 76.8 (d, 5’”), 70.2 (t, 6’”), 104.5 (d, 1””), 75.3 (d, 2””), 76.3 (d, 3””), 71.9 (d, 4””), 81.0 (d, 5””), 62.8 (t, 6””).

a

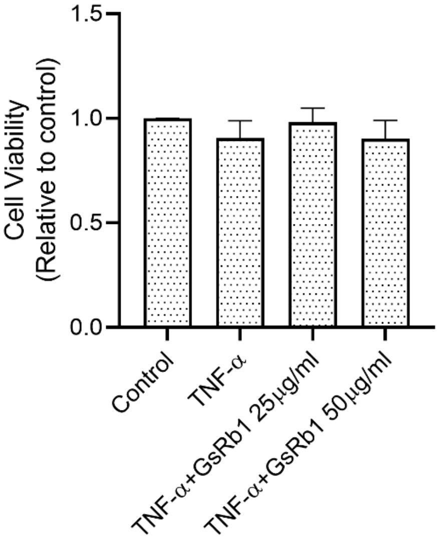

b

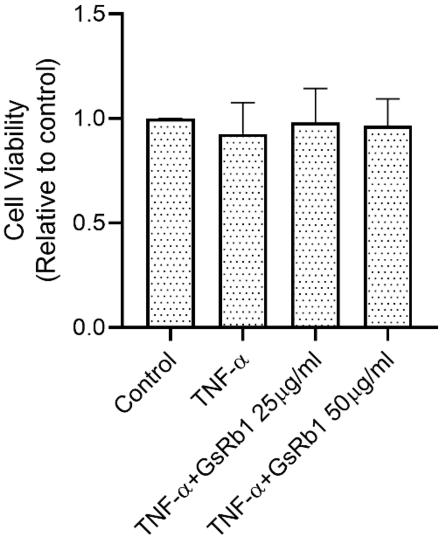

Figure S6: Effects of GsRb1 on the cell viability of H9c2 (a) and HepG-2 (b) cells. H9c2 or HepG-2 cells were pretreated with GsRb1 (25, 50μg/ml) for 24hrs, then the cells were stimulated by recombinant rat TNF-α (10ng/ml) for another 72hrs. After the treatment, the cells were incubated with 0.5mg/ml MTT solution for 1hr at 37°C. And then 200μl isopropyl alcohol (IPP) was added after the medium was discarded. After 10 min of incubation, the absorbance was measured at 570nm by using a microplate reader (BioRad). Results are shown as mean ±SD from 4 independent experiments.

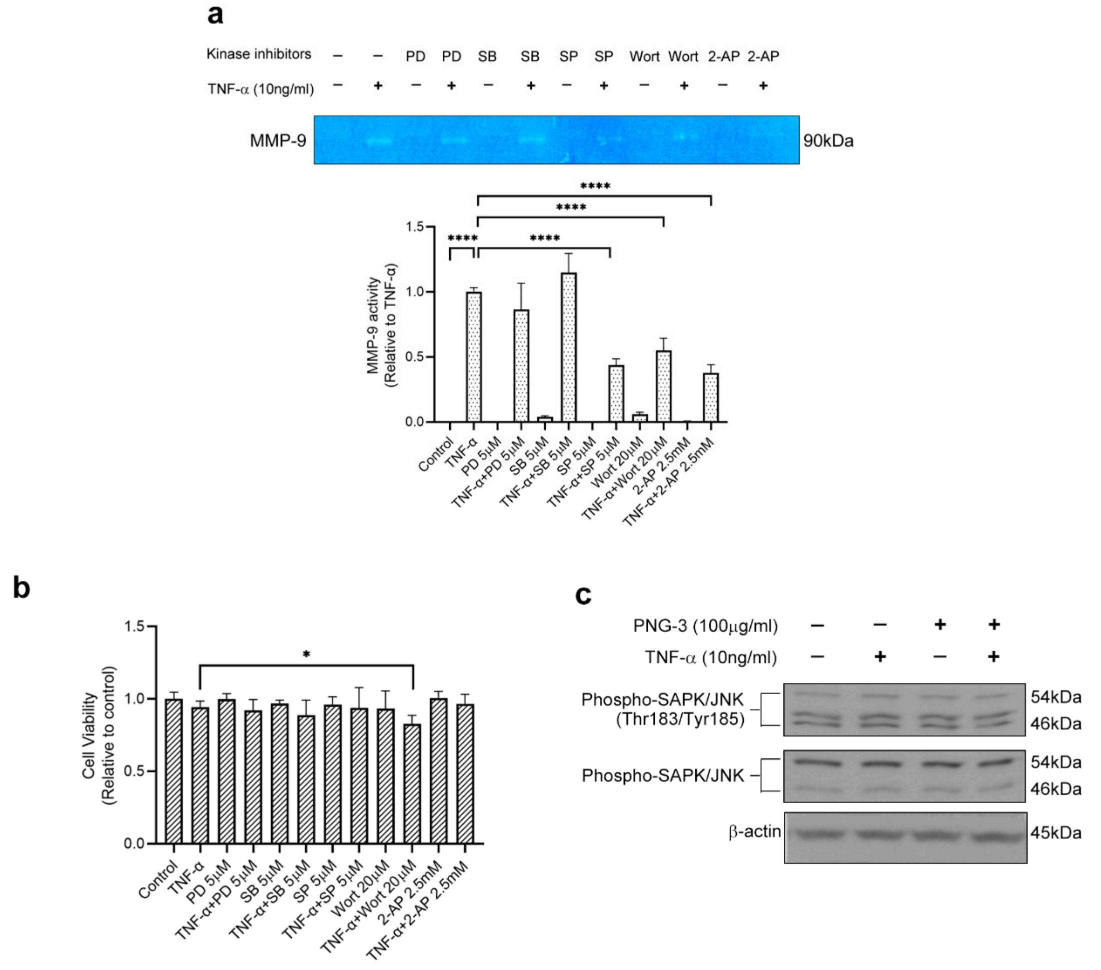

Figure S7: Underlying mechanism involved in TNF-α-indued MMP-9 activation in H9c2 cells. a. Effect of different kinase inhibitors on TNF-α-induced MMP-9 protein activity in H9c2 cells. Before the treatment of TNF-α (10ng/ml) for 72hrs, H9c2 cell (1×10^5) was pretreated for 1hr with PD 98059 (5μM), SB 203580 (5μM), SP 600125 (5μM), Wortmannin (20μM) and 2-AP (2.5mM), respectively. The fold induction of MMP-9 activity was normalized with that of the TNF-α only treated cells. Results of bar graphs are shown as mean ±SD from 4 independent experiments, ****p<0.0001. PD, PD 98059; SB, SB 2035820; SP, SP 600125; Wort, Wortmannin. **b.** Effect of different kinase inhibitors on the cell viability in H9c2 cells. N=4, *p<0.05. **c.** Effect of PNG-3 on the phosphorylation of JNK in TNF-α-induced H9c2 cell. N=3.

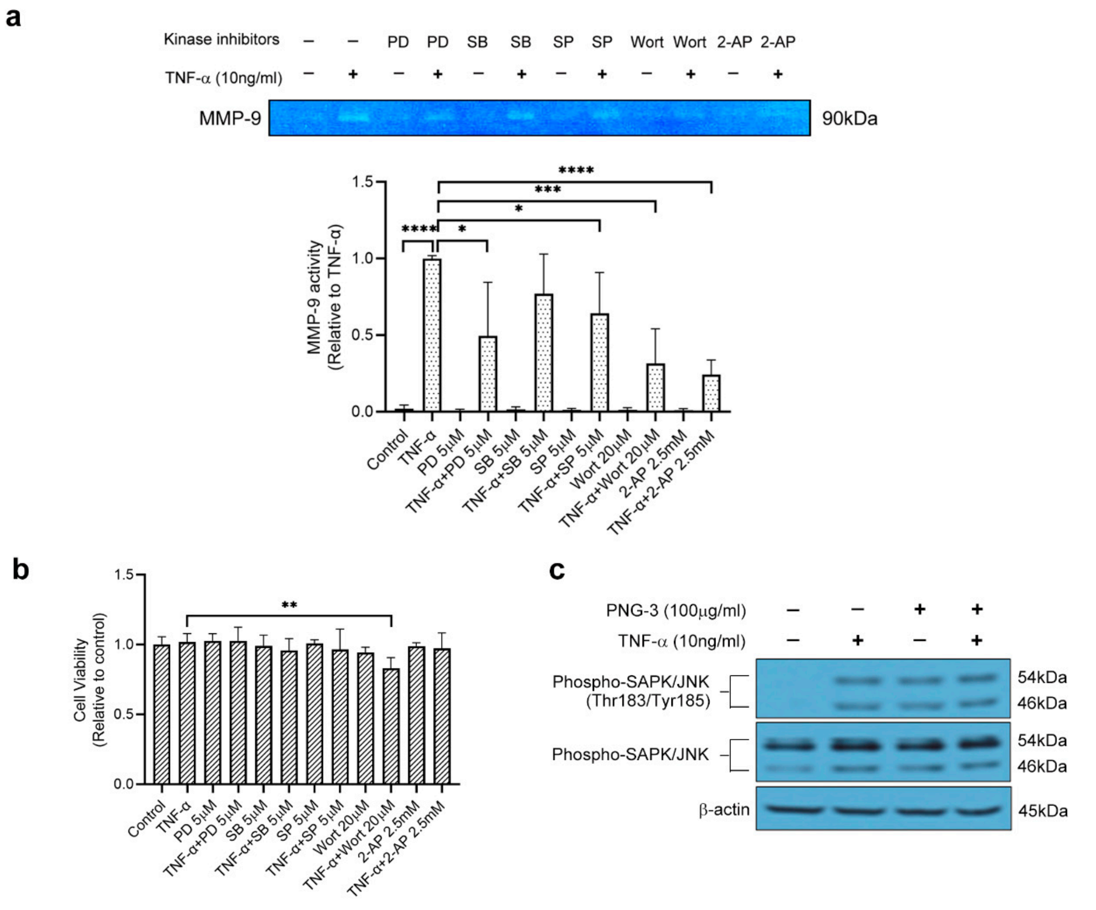

Figure S8: Underlying mechanism involved in TNF-α-indued MMP-9 activation in HepG-2 cells. a. Effect of different kinase inhibitors on the TNF-α induced MMP-9 protein activity in HepG-2 cell. Before the treatment of TNF-α (10ng/ml) for 72hrs, HepG-2 cell (1×10^5) was pretreated for 1hr with PD 98059 (5μM), SB 203580 (5μM), SP 600125 (5μM), Wortmannin (20μM) and 2-AP (2.5mM), respectively. The fold induction of MMP-9 activity was normalized with that of the TNF-α-only treated cells. Results of bar graphs are shown as mean ±SD from 4 independent experiments. **p*< 0.05, ****p*<0.001, *****p*<0.0001. PD, PD 98059; SB, SB 2035820; SP, SP 600125; Wort, Wortmannin. **b.** Effect of different kinase inhibitors on the cell viability in HepG-2 cell. N=4, ***p*< 0.01. **c.** Effect of PNG-3 on the phosphorylation of JNK in TNF-α-induced H9c2 cell. N=3.

Table S1: Summary of MMP-inhibitory saponins in *Panax notoginseng.*

Compound Name	Cell used	In vitro/ ex vivo Inducer	Functions	Mechanisms	References
Ginsenoside Rb1	Rat Hepatic Stellate Cells (HSC-T6)	H_2O_2	↓MMP-2<br>↓TIMP-1	----	(1)
	Human keratinocytes	----	↑MMP-2<br>↑MMP-9	S1P/S1P receptor(s)/ERK1/2 /NF-κB	(2)
	human umbilical vein endothelial cells (HUVECs)	TNF-α	↓MMP-2<br>↓MMP-9	JNK, p38 MAPK/ NF-κB	(3)
Ginsenoside Rh1	Human hepatocellular carcinoma (HepG2)	Phorbol myristate acetate (PMA)	↓MMP-1	↓MAPKs-ERK1/2 p38 MAPK,<br>JNK1/2<br>Phosphorylation	(4)
	human colorectal cancer cell line SW620	----	↓MMP-1<br>↓MMP-3<br>↑TIMP-3	↓MAPKs-ERK1/2 p38 MAPK,<br>JNK1/2<br>Phosphorylation	(5)
Ginsenoside Rb2	Human endometrial cancer cell lines<br>(Ishikawa;<br>HHUA;<br>HEC-1-A)	----	↓MMP-2<br>→TIMP-1<br>→TIMP-2	----	(6)
	human dermal fibroblasts	UV-B	↓MMP-2	----	(7)
Ginsenoside Rd	Human hepatocellular carcinoma (HepG2)	----	↓MMP-1<br>↓MMP-2<br>↓MMP-7	↓MAPKs-ERK1/2<br>P38 MAPK phosphorylation	(8)

				↓AP-1 activation	
	Human keratinocytes	H_2O_2	↓MMP-1	cAMP	(9)
20(S)-Ginsenoside Rg3	TC28a2 Human Chondrocytes	TNF-α	↓MMP-9	SIRT1/PGC-1α/SIRT3/p38 MAPK/NF-κB	(10)
	Human ovarian cancer (SKOV-3)	----	↓MMP-9	----	(11)
	Murine macrophage cell (RAW264.7)	LPS	↓MMP-9	----	(12)
	Isolated human chondrocytes	IL-1β	↓MMP-1 ↓MMP13	----	(13)
20(R)-Ginsenoside Rg3	Human unbilical vein endothelial cell (HUVEC)	----	↓MMP-2 ↓MMP-9	----	(14)
Ginsenoside Rh2 (steamed Panax notoginseng)	Human lung adenocarcinoma A549, H1299	----	↓MMP-9	mir-491	(15)
	Human astroglioma (U87MG; U373MG)	PMA	↓MMP-1 ↓MMP-3 ↓MMP-9 ↓MMP14 →MMP-2	↓MAPKs-ERK1/2 P38 MAPK, JNK1/2 Phosphorylation ↓NF-κB,AP-1 activity	(16)
Total saponin of Panax notoginseng	Rat,mice, rabbit	*in vivo*	↓MMP-9	ATF3/MAP2K3/p38 MAPK/NFκ B	(17-19)

Supplemental References

1. Lo YT, Tsai YH, Wu SJ, Chen JR, Chao JC. Ginsenoside Rb1 inhibits cell activation and liver fibrosis in rat hepatic stellate cells. J Med Food. 2011;14(10):1135-43.
2. Shin KO, Choe SJ, Uchida Y, Kim I, Jeong Y, Park K. Ginsenoside Rb1 Enhances Keratinocyte Migration by a Sphingosine-1-Phosphate-Dependent Mechanism. J Med Food. 2018;21(11):1129-36.
3. Zhou P, Lu S, Luo Y, Wang S, Yang K, Zhai Y, et al. Attenuation of TNF-alpha-Induced Inflammatory Injury in Endothelial Cells by Ginsenoside Rb1 via Inhibiting NF-kappaB, JNK and p38 Signaling Pathways. Front Pharmacol. 2017;8:464.
4. Yoon JH, Choi YJ, Lee SG. Ginsenoside Rh1 suppresses matrix metalloproteinase-1 expression through inhibition of activator protein-1 and mitogen-activated protein kinase signaling pathway in human hepatocellular carcinoma cells. Eur J Pharmacol. 2012;679(1-3):24-33.
5. Lyu X, Xu X, Song A, Guo J, Zhang Y, Zhang Y. Ginsenoside Rh1 inhibits colorectal cancer cell migration and invasion in vitro and tumor growth in vivo. Oncol Lett. 2019;18(4):4160-6.
6. Fujimoto J, Sakaguchi H, Aoki I, Toyoki H, Khatun S, Tamaya T. Inhibitory effect of ginsenoside-Rb2 on invasiveness of uterine endometrial cancer cells to the basement membrane. Eur J Gynaecol Oncol. 2001;22(5):339-41.
7. Oh SJ, Kim K, Lim CJ. Ginsenoside Rb2 Attenuates UV-B Radiation-Induced Reactive Oxygen Species and Matrix Metalloproteinase-2 through Upregulation of Antioxidant Components in Human Dermal Fibroblasts. Pharmacology. 2015;96(1-2):32-40.
8. Yoon JH, Choi YJ, Cha SW, Lee SG. Anti-metastatic effects of ginsenoside Rd via inactivation of MAPK signaling and induction of focal adhesion formation. Phytomedicine. 2012;19(3-4):284-92.
9. Kim WK, Song SY, Oh WK, Kaewsuwan S, Tran TL, Kim WS, et al. Wound-healing effect of ginsenoside Rd from leaves of Panax ginseng via cyclic AMP-dependent protein kinase pathway. Eur J Pharmacol. 2013;702(1-3):285-93.
10. Ma CH, Chou WC, Wu CH, Jou IM, Tu YK, Hsieh PL, et al. Ginsenoside Rg3 Attenuates TNF-alpha-Induced Damage in Chondrocytes through Regulating SIRT1-Mediated Anti-Apoptotic and Anti-Inflammatory Mechanisms. Antioxidants (Basel). 2021;10(12).
11. Xu TM, Cui MH, Xin Y, Gu LP, Jiang X, Su MM, et al. Inhibitory effect of ginsenoside Rg3 on ovarian cancer metastasis. Chin Med J (Engl). 2008;121(15):1394-7.
12. Shin YM, Jung HJ, Choi WY, Lim CJ. Antioxidative, anti-inflammatory, and matrix metalloproteinase inhibitory activities of 20(S)-ginsenoside Rg3 in cultured mammalian cell lines. Mol Biol Rep. 2013;40(1):269-79.
13. So MW, Lee EJ, Lee HS, Koo BS, Kim YG, Lee CK, et al. Protective effects of ginsenoside Rg3 on human osteoarthritic chondrocytes. Mod Rheumatol. 2013;23(1):104-11.

14. Yue PY, Wong DY, Wu PK, Leung PY, Mak NK, Yeung HW, et al. The angiosuppressive effects of 20(R)- ginsenoside Rg3. Biochem Pharmacol. 2006;72(4):437-45.
15. Chen Y, Zhang Y, Song W, Zhang Y, Dong X, Tan M. Ginsenoside Rh2 Inhibits Migration of Lung Cancer Cells under Hypoxia via mir-491. Anticancer Agents Med Chem. 2019;19(13):1633-41.
16. Kim SY, Kim DH, Han SJ, Hyun JW, Kim HS. Repression of matrix metalloproteinase gene expression by ginsenoside Rh2 in human astroglioma cells. Biochem Pharmacol. 2007;74(11):1642-51.
17. Liu G, Wang B, Zhang J, Jiang H, Liu F. Total panax notoginsenosides prevent atherosclerosis in apolipoprotein E-knockout mice: Role of downregulation of CD40 and MMP-9 expression. J Ethnopharmacol. 2009;126(2):350-4.
18. Wu L, Zhang W, Tang YH, Li H, Chen BY, Zhang GM, et al. Effect of total saponins of "panax notoginseng root" on aortic intimal hyperplasia and the expressions of cell cycle protein and extracellular matrix in rats. Phytomedicine. 2010;17(3-4):233-40.
19. Ma RF, Chen G, Li HZ, Zhang Y, Liu YM, He HQ, et al. Panax Notoginseng Saponins Inhibits Ventricular Remodeling after Myocardial Infarction in Rats Through Regulating ATF3/MAP2K3/p38 MAPK and NF kappa B Pathway. Chin J Integr Med. 2020;26(12):897-904.
